# Characteristics of Herbal Medicine Users and Adverse Events Experienced in South Korea: A Survey Study

**DOI:** 10.1155/2017/4089019

**Published:** 2017-04-10

**Authors:** Soobin Jang, Kyeong Han Kim, Seung-Ho Sun, Ho-Yeon Go, Eun-Kyung Lee, Bo-Hyoung Jang, Yong-Cheol Shin, Seong-Gyu Ko

**Affiliations:** ^1^Department of Preventive Medicine, College of Korean Medicine, Kyung Hee University, 26 Kyungheedae-ro, Dongdaemun-gu, Seoul 02447, Republic of Korea; ^2^KM Fundamental Research Division, Korea Institute of Oriental Medicine, 1672 Yuseongdae-ro, Yuseong-gu, Daejeon 34054, Republic of Korea; ^3^Department of Preventive Medicine, College of Korean Medicine, Woosuk University, 61 Seonneomeo 3-gil, Wansan-gu, Jeonju, Jeollabuk-do 54986, Republic of Korea; ^4^Department of Korean Internal Medicine, Sangji University Korean Medicine Hospital, 283 Woosan-dong, Wonju 26338, Republic of Korea; ^5^Department of Korean Internal Medicine, College of Korean Medicine, Semyung University, 579 Sinwoul-dong, Jecheon, Chungcheongbuk-do 27136, Republic of Korea

## Abstract

*Background.* This survey aimed to investigate the characteristics of users and nonusers of herbal medicine and the adverse events experienced due to herbal medicines in South Korea.* Methods.* The questionnaire consisted of safety, using experience, using type, usage and nonusage reason, purchase location, and adverse events of herbal medicine. The survey was administered by online.* Results.* Of the total 1,134 respondents, 726 (64.0%) considered herbal medicine safe, and 693 (61.1%) answered that they have taken herbal medicines within the past year. Most common place to purchase them was “TKM hospital or clinic” (63.6%), and most participants (72.2%) took a decoction from a TKM institution. The biggest reason for taking them was for “health improvement” (57.3%), and the reasons for not using them was “medication not necessary” (63.7%). Among those who took herbal medicines, 46 experienced adverse events, and the most frequently reported symptoms were digestive disorders (52.2%). Of the 46 participants who experienced adverse events, 20 (43.5%) were treated by TKM doctors.* Conclusions.* This study suggests that regulation of herbal medicines is needed in order to resolve problems related to the safety of herbal medicines.

## 1. Background

The global herbal medicine market has grown every year, and the total estimated retail sales of herbal supplements in the United States reached almost $7 billion after increasing by 7.5% in 2015 [1]. The sales are anticipated to further grow to reach $5 trillion by 2050 [2].

As the interest in herbal medicines increases, there is a growing need to ensure their safety. The goal of the World Health Organization (WHO) Traditional Medicine Strategy is to promote the safe and effective use of Traditional and Complementary Medicine (T&CM). Regulations on the safety of herbal medicine have increased in order to achieve this goal. The numbers of policies and regulations to ensure the safety and efficacy of T&CM were 69 and 119, respectively [3]. According to the Report on Usage and Consumption of Korean Medicines 2011 [4], approximately 22.4% of people who have experienced Korean Medicine hospitals or clinics answered that ensuring the safety of herbal medicines would lead to future improvements in Korean Medicine.

South Korea is a country with a long history of herbal medicine usage. However, until recently, there has not been a systematic report of adverse events, which would be the foundation of enhancing safety. According to the data of Korean Regional Pharmacovigilance Centers in 2007, there was only one case of adverse drug reaction reported from herbs among the observed 1,418 cases [5]. As a result of self-investigation of a university hospital, 28 adverse drug reactions occurred from herbal medicines in one Korean Medicine Hospital in South Korea from January 1, 2008, to February 29, 2012 [6].

This study surveyed in detail the characteristics of consumers in Korea and the adverse events from herbal medicines, as well as the perceived safety of products. The objective of this study was to investigate Korean consumers' usage patterns with respect to herbal medicines and to provide research-based evidence for enhancing their safety.

## 2. Methods

### 2.1. Study Design and Setting

This study was a survey of the characteristics of herbal medicine users in South Korea. The survey was conducted by Macromill Embrain (http://www.embrain.com), which is a professional survey research company that manages about 1,180,000 online research panels in South Korea. The company recruited the participants with consideration of the age and sex distributions and informed them that responses had to be filled out for all our questions in the questionnaire. The participants were enrolled on a voluntary basis and there was no refusal rate. The survey was conducted anonymously between October 1 and 31, 2015.

### 2.2. Participants

There was no special method for determining the sample size; we only sought to have as many people as possible complete the survey during the survey period. The minimum number of participants was determined to be 1,000, with additional recruitment on-going until the end of the study period. The participants were stratified based on gender and age to get the status of general population. Those under 20 years old and over 70 years old were excluded.

### 2.3. Questionnaire

The questionnaire was developed by five traditional Korean medicine (TKM) experts who discussed and selected investigation items. A draft questionnaire was developed through two rounds of review, placing emphasis on the easy comprehension of the questionnaire to gear to the general population. The experts examined face reliability as well as readability of the questionnaire. Then, a pilot test was conducted that targeted 10 people who were not medical practitioners. A group of experts collected feedback and completed the final version of the questionnaire.

The questionnaire consisted of two categories: (1) questions related to the herbal medicines usage over the past year and (2) questions related to adverse events experienced relating to the herbal medicines. The questionnaire is shown in Supplement 1 (in Supplementary Material available online at https://doi.org/10.1155/2017/4089019).

### 2.4. Study Variables

The detailed variables are as follows:

1. Demographic information: sex, age, occupation, and education level
2. Usage patterns: opinion on safety of herbal medicines, experiences related to taking herbal medicines, places from which the herbal medicines were purchased, the types of herbal medicines used, reasons for taking herbal medicines, and reasons for not taking herbal medicines
3. Adverse events: experiences of adverse events relating to herbal medicines, types of adverse events, whether adverse events were reported, to which institutions the adverse events were reported, reasons for not reporting adverse events, how to deal with adverse events, and opinions on herbal medicines after experiencing adverse events

### 2.5. Statistical Analyses

A frequency analysis was performed for all variables. The chi-squared test was also employed in order to determine differences by sex, age, occupation, and education level. IBM SPSS ver. 18.0 (IBM Co., Armonk, NY, USA) was used for analysis.

### 2.6. Ethical Considerations

All participants were briefed with an explanation of the study's purpose prior to the initiation of the survey. Only those who voluntarily agreed to participate and to have their data collected to be published were enrolled in the study. This survey was conducted anonymously. The entire survey process was approved by the Institutional Review Board of Kyung Hee University (IRB number KHSIRB1-15-039).

## 3. Results

### 3.1. Basic Characteristics

There were total of 1,134 respondents, consisting of 591 (52.1%) men and 543 (47.9%) women.  Table 1 presents the distribution of the participants' sex, age, occupation, and education level. The age distribution was as follows: 209 (18.4%) were 20–29 years old, 237 (20.9%) were 30–39 years old, 277 (24.4%) were 40–49 years old, 253 (22.3%) were 50–59 years old, and 158 (13.9%) were 60–69 years old. Office worker was the most common occupation (34.6%), and most participants (79.7%) had a university degree.

Of the 1,134 respondents, there were 693 (61.1%) who had taken herbal medicines within the past year and 441 (38.9%) who had not. There was no difference in demographic factors between users and nonusers of herbal medicines (Table 2).

### 3.2. Opinion on Safety of Herbal Medicines

Of the total 1,134 participants, 726 (64.0%) people responded that herbal medicine is safe and the remaining 408 (36.0%) people considered herbal medicine unsafe. Women tended to distrust the safety of herbal medicines more compared to men, and those over the age of 50 were more skeptical of herbal medicine (Table 3).

### 3.3. Usage Patterns of Herbal Medicines

The most common place to purchase herbal medicines was TKM hospital or clinic (63.6%). Pharmacy (17.0%), traditional herb market (17.0%), health food store (14.6%), oriental pharmacy (12.8%), home shopping (11.0%), and hypermarket (11.0%) were reported as other places to purchase them. The most predominantly used type of herbal medicines was a decoction from TKM institutions (72.2%). Other types of herbal medicines were crude herbs, which are mainly used in food or tea (35.8%), health food (28.6%), national insurance-covered herbal medicines from TKM institutions (15.3%), national insurance-covered herbal medicines from pharmacies (15.0%), and others (0.8%) (Table 4).

**Figure 1:**
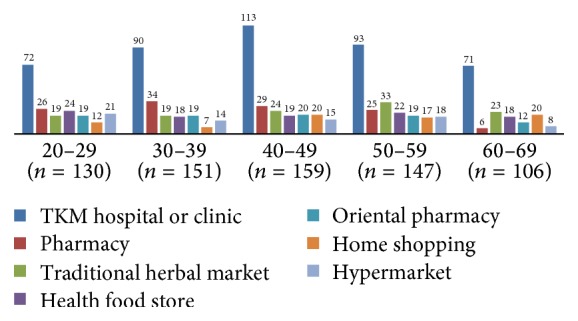
Location where herbal medicines were purchased according to age groups.

**Figure 2:**
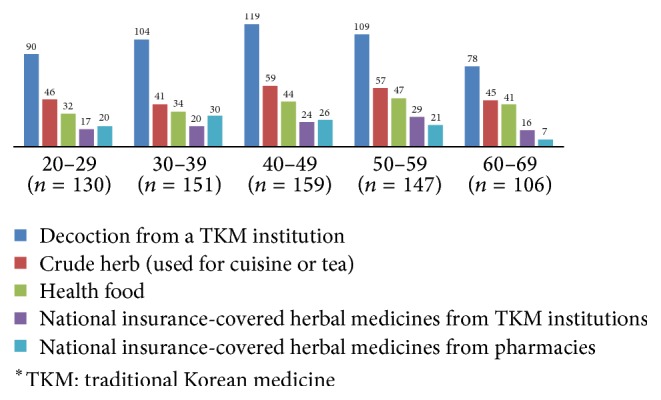
Types of herbal medicines according to age groups.

**Figure 3:**
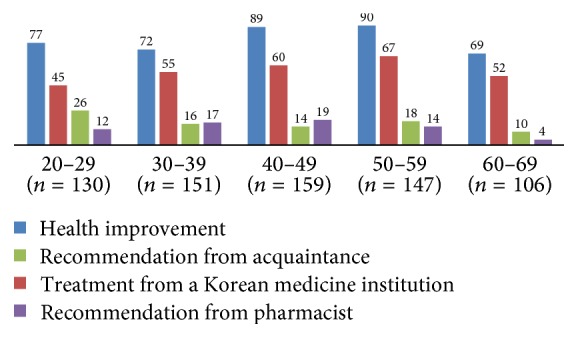
Reasons for taking herbal medicines according to age groups.

The reasons for taking the medication were as follows: 57.3% for “health improvement,” 40.3% for “treatment in KM hospitals or clinics,” 34.8% due to “recommendation from acquaintance,” 9.5% due to “recommendation from a pharmacist,” and others. The reasons for not taking herbal medicines were “medication was not necessary” (63.7%), “uncertainty of origins” (35.4%), “expensive prices” (25.9%), “anxiety related to the possibility of harmful substances” (25.9%), “anxiety related to the possibility of adverse events” (23.8%), and others (Table 4). Figures 1–3 present the usage patterns and reasons for taking of herbal medicines according to age groups in detail. There were no remarkable differences by age.

### 3.4. Adverse Events and Their Reporting

Of the 693 participants who have taken herbal medicines within the past year, 46 (6.6%) responded that they had experienced adverse events from herbal medicines. The most common symptom was digestive disorders (52.2%), followed by skin disorders (34.8%) and nervous disorders (23.9%) (Table 5).

After experiencing an adverse event, 20 participants (43.5%) were treated by KM doctors, 13 (28.5%) did not take any action, and 12 (26.1%) requested a refund. Seventeen participants (37.0%) felt that expert counselling may be needed after experiencing adverse events, and 14 (30.4%) responded that drugs can have adverse events and that they would continue taking herbal medicines. However, 13 people (28.3%) responded that they cannot trust herbal medicines anymore and they would not continue taking herbal medicines. Of the 46 respondents who experienced adverse events, 14 (30.4%) reported their adverse events and 20 (43.5%) did not because they had little information regarding to whom the report should be made (Table 6).

## 4. Discussion

This study described the basic characteristics of those who had taken herbal medicines and those who had not taken them, the places that herbal medicines were purchased, the reasons for taking or not taking herbal medicine, and adverse events experienced due to herbal medicines and how the adverse events were addressed. In previous studies [7, 8], elderly people tended to visit TKM institutions more frequently than younger people. However, age was not a factor that affected taking herbal medicines in this survey.

There have been several surveys in the past demonstrating consumers' opinions about the safety of herbal medicines [9–11]. According to surveys of Serbia [9] and Saudi Arabia [10], 73.3% (211) of Serbian respondents and 81.2% (239) of Saudi Arabian respondents considered that the use of herbal medicines and herbal dietary supplements is harmless, respectively. Meanwhile, only 12.1% (88) of Lebanese respondents perceived that herbal products sold in Lebanon are pure [11].

Most respondents purchased herbal medicines from TKM institutions (Table 4). This observation can be explained by the Korean health system. South Korea has adopted a dual healthcare system in which both Western medicine and TKM are permitted as legal medical care [12]. Herbal-drugs are separated from herbal supplements and are prescribed by TKM practitioners or sold over-the-counter in pharmacies. Herbal supplements, such as red ginseng, are sold in pharmacies, hypermarkets, or through home shopping channels. Traditional herb markets and health food stores generally sell crude herbs or self-made decoctions. Oriental pharmacies can provide 100 popular herbal medications without TKM practitioners' prescriptions [13].

Regarding the type of herbal medicines, a decoction from a TKM institution was the most frequently used, reflecting the preference of Koreans (Table 4). Korean people may recognise that a decoction is a typical herbal medicine that is more effective than other formulations, such as powders, pills, and capsules. On the other hand, in Japan, the proportion of the market of herbal medicines covered by insurance is large, and production costs of insured herbal medicines account for 84.2% of the entire herbal medicine market [14].

The main reasons for taking herbal medicines included “health improvement” and “treatment in TKM hospitals or clinics” (Table 4). Herbal medicine is recognised as a tool for both preventive medicine and disease treatment in South Korea. Meanwhile, traditional medicine is still one of the primary sources of health care in Africa and some other developing countries, and it is used as complementary therapy in North America and many European countries [3]. In South Korea, both uses are well-balanced thanks to the health system and cultural influences.

This study also analysed the usage patterns of difference by age (Figures 1–3). There is no noticeable outcome, though those 60–69 tend to use crude herb and health food more. It may affect their negative perception on safety of herbal medicines (Table 3). Crude herb is not purified and its safety is not proven. It also includes roots, leaves, and flowers that have been taken from the wild; accordingly, the safety of crude herb can be suspicious.

Of the 1,134 respondents, 441 (38.9%) had not taken herbal medicines in the past year (Table 2). Among the reasons for not taking herbal medicine, “uncertainty of origins,” “anxiety related to the possibility of harmful substances,” “anxiety related to the possibility of adverse events,” and “distrust of expiry date” were due to disbelief in the safety of herbal medicines. It is necessary to improve the safety of herbal medicines in order for the herbal medicine market to grow. In South Korea, regulations on manufacturing and quality control of herbal medications were established in 2012, and they became fully mandatory in 2015 [15]. Health food is subject to the Good Manufacturing Practices (GMP) of the Ministry of Food and Drug Safety (MFDS) [16]. However, there is no safety control system for herbal medicines distributed via other routes. In Japan, unlike in South Korea, herbal medicines are divided into 210 over-the-counter Kampo products, crude drugs, and Kampo extracts and Western traditional herbal products, which are separately managed according to the national system [17].

Adverse events from herbal medicines reported by 46 participants (6.6% of herbal medicines users) primarily included digestive, skin, and nervous disorders (Table 5). Liver toxicity of herbal medicines is controversial when it comes to the safety of herbal medicine [18]. However, only 4 cases of liver disorders were reported among the total 77 cases. According to previous studies [6, 19], the most frequently reported adverse drug reactions in a single hospital were gastrointestinal disorders and skin reactions, similar to the results in our study.

Although the Korean adverse drug reaction surveillance system was established in 1988 [20], it is not appropriate for reporting adverse events due to herbal medicines. Since 2012, adverse reactions from approved herbal-drugs have been reported to the Korea Adverse Event Reporting System (KAERS). However, not every herbal medicine is registered in that system, and the formulation of decoctions has not been applicable. The problem about national pharmacovigilance system of South Korea was also raised in prior study [21]. On the other hand, in Taiwan, the compositions and formulations of herbal medicines are included when reporting adverse drug reactions [22].

After experiencing adverse events, the majority of respondents (20; 43.5% of adverse event experiencers) visited TKM practitioners, and 17 (37.0%) felt that expert counsel would be necessary (Table 6). These results suggest that the role of TKM practitioners is important when adverse events occur. Moreover, only 14 people (30.4% of adverse events experiencers) reported their adverse events, and only one person reported it to the Korea Institute of Drug Safety and Risk Management (KIDS) correctly. Therefore, both TKM practitioners and consumers need to receive appropriate education for responding to adverse events due to herbal medicines. Additionally, the reliability on herbal medicines was reduced for 13 respondents after they experienced adverse events (28.3% of adverse events experiencers), and they responded that they would not take any other herbal medicines. Such behaviours may lead to a decreased consumption of herbal medicines; consequently, the safety of herbal medicines is vitally important.

There are limitations of this survey study. Firstly, a recall bias may exist because this study was based on a retrospective survey. Secondly, there is a possibility of response bias because the participants are rather highly educated. This is because having recruited the participants through an online research company and the sample may not be representative of the general population. Lastly, the perception of the range of herbal medicines varies among Koreans. Some people only recognise herbal medicines from TKM institutions as herbal medicines, while others take into account every type of herb.

Nonetheless, this study is meaningful in that there are no previous surveys to date that systematically investigated experiences and opinions about herbal medicines. This survey, unlike other consumer surveys, included not only herbal medicines users but also nonusers as participants, which increased the representativeness of the general population.

## 5. Conclusions

This survey analysed the usage of herbal and medicinal products in South Korea. This study showed the demographic differences between herbal medicine users and nonusers, opinions on safety of herbal medicines, experiences of using herbal medicines, and adverse events experienced from using herbal medicines. The major reasons for not taking herbal medicines were based on a disbelief in their safety. Therefore, it is important to ensure not only the efficacy but also the safety of herbal medicines in order to expand herbal product markets. Specific regulations on herbal medicines are needed to resolve problems with their origins, possibility of containing harmful substances, and the expiry date.

## Supplementary Material

The questionnaire aimed to investigate the usage experience of herbal medicines of general populations. It consists of two categories: (1) questions related to the herbal medicines usage over the past year and (2) questions related to adverse events experienced relating to the herbal medicines.

## Figures and Tables

**Table 1:** Basic characteristics of respondents.

Demographic characteristics	*n*	%
Sex		
Men	591	52.1
Women	543	47.9
Age (years)		
20–29	209	18.4
30–39	237	20.9
40–49	277	24.4
50–59	253	22.3
60–69	158	13.9
Occupation		
Executives professionals	214	18.9
Office workers	392	34.6
Service sales workers	83	7.3
Agriculture, forestry, and fishery workers	4	0.4
Craft mechanical workers	41	3.6
Simple labourers	15	1.3
Self-employed, part-time employees, and freelancers	25	2.2
Students, housewives, and unemployed	360	31.7
Level of education		
Middle school	9	1.1
High school	218	19.2
College	784	69.1
Graduate school	120	10.6

Total	1134	100.0

**Table 2:** Basic characteristics of herbal medicines users and nonusers.

Demographic characteristics	Users (*n* = 693, 61.1%)	Nonusers (*n* = 441, 38.9%)	^*∗*^ *P* value
Sex			
Men	361 (52.1)	230 (52.2)	1.000
Women	332 (47.9)	211 (47.8)	
Age (years)			
20–29	130 (18.8)	79 (17.9)	0.221
30–39	151 (21.8)	86 (19.5)	
40–49	159 (22.9)	118 (26.8)	
50–59	147 (21.2)	106 (24.0)	
60–69	106 (15.3)	52 (11.8)	
Occupation			
Executives professionals	146 (21.1)	68 (15.4)	0.229
Office workers	244 (35.2)	148 (33.6)	
Service sales workers	45 (6.5)	38 (8.6)	
Agriculture, forestry, and fishery workers	2 (0.3)	2 (0.5)	
Craft mechanical workers	23 (3.3)	18 (4.1)	
Simple labourers	7 (1.0)	8 (1.8)	
Self-employed, part-time employees, and freelancers	15 (2.2)	10 (2.3)	
Students, housewives, and unemployed	211 (30.4)	149 (33.8)	
Level of education			
Middle school	6 (0.9)	6 (1.3)	0.172
High school	119 (17.2)	99 (22.4)	
College	490 (70.7)	294 (66.7)	
Graduate school	78 (11.3)	42 (9.5)	

All data are in *n* (%).

^*∗*^Chi-square test was performed.

**Table 3:** Opinion on safety of herbal medicines.

	Herbal medicine is safe (*n* = 726, 64.0%)	Herbal medicine is not safe (*n* = 408, 36.0%)	^*∗*^ *P* value
Sex			
Men	430 (59.2)	161 (39.5)	>0.001
Women	296 (40.8)	247 (60.5)	
Age (years)			
20–29	154 (21.2)	55 (13.5)	>0.001
30–39	166 (22.9)	71 (17.4)	
40–49	176 (24.2)	101 (24.8)	
50–59	148 (20.4)	105 (25.7)	
60–69	82 (11.3)	76 (18.6)	
Occupation			
Executives professionals	145 (20.0)	69 (16.9)	0.142
Office workers	252 (34.7)	140 (34.3)	
Service sales workers	52 (7.2)	31 (7.6)	
Agriculture, forestry, and fishery workers	4 (0.6)	0	
Craft mechanical workers	32 (4.4)	9 (2.2)	
Simple labourers	11 (1.5)	4 (1.0)	
Self-employed, part-time employees, and freelancers	14 (1.9)	11 (2.7)	
Students, housewives, and unemployed	216 (29.8%)	144 (35.3)	
Level of education			
Middle school	10 (1.4)	2 (0.4)	0.610
High school	138 (19.0)	80 (19.6)	
College	499 (68.7)	285 (69.9)	
Graduate school	79 (10.9)	41 (10.0)	

All data are in *n* (%).

^*∗*^Chi-square test was performed.

**Table 4:** Patterns of herbal medicine use and reasons for taking or not taking herbal medicines.

Question (number of respondents)	Response	*n* (%)
Have you taken herbal medicines in the past year? (*n* = 1134)	Yes	693 (61.1)
	No	441 (38.9)

Location where herbal medicines were purchased^*∗*^ (*n* = 693)	TKM hospital or clinic	441 (63.6)
	Pharmacy	120 (17.0)
	Traditional herbal market	118 (17.0)
	Health food store	101 (14.6)
	Oriental pharmacy	89 (12.8)
	Home shopping	76 (11.0)
	Hypermarket	76 (11.0)
	Other	8 (1.1)

Types of herbal medicines^*∗*^ (*n* = 693)	Decoction from a TKM institution	500 (72.2)
	Crude herb (used for cuisine or tea)	248 (35.8)
	Health food	198 (28.6)
	National insurance-covered herbal medicines from TKM institutions	106 (15.3)
	National insurance-covered herbal medicines from pharmacies	104 (15.0)
	Other	6 (0.8)

Reasons for taking herbal medicines^*∗*^ (*n* = 693)	Health improvement	397 (57.3)
	Treatment from a Korean medicine institution	279 (40.3)
	Recommendation from acquaintance	84 (12.1)
	Recommendation from pharmacist	66 (9.5)
	Other	6 (0.7)

Reasons for not taking herbal medicines^*∗*^ (*n* = 441)	Medication not necessary	281 (63.7)
	Uncertainty of origins	156 (35.4)
	Expensive prices	114 (25.9)
	Anxiety about possible harmful substances	105 (23.8)
	Anxiety about possible adverse events	59 (13.4)
	Disbelief regarding expiry date	41 (9.3)
	No effectiveness	39 (8.8)
	Other	7 (1.6)

^*∗*^Multiple responses possible; TKM: traditional Korean medicine.

**Table 5:** The number of adverse events due to herbal medicines.

Question (number of respondents)	Response	*n* (%)
Have you experienced adverse events from herbal medicines in the past year? (*n* = 693)	Yes	46 (6.6)
	No	647 (93.4)

What types of adverse events have you experienced?^*∗*^ (*n* = 46)	Digestive system	24 (52.2)
	Skin	16 (34.8)
	Nervous disorder	11 (23.9)
	Systemic disorder	6 (13.0)
	Liver	4 (8.7)
	ENT and eye	3 (6.5)
	Cardiovascular system	3 (6.5)
	Circulatory system	2 (4.3)
	Kidney	2 (4.3)
	Urinary system	2 (4.3)
	Musculoskeletal system	2 (4.3)
	Respiratory system	2 (4.3)

^*∗*^Multiple responses possible; ENT, ear, nose, and throat.

**Table 6:** Behaviours and opinions related to herbal medicine after experiencing adverse events.

Question (number of respondents)	Response	*n* (%)
Did you report adverse events? (*n* = 46)	Yes	14 (30.4)
	No	32 (69.6)

To whom did you report adverse events?^*∗*^ (*n* = 14)	TKM institution	10 (71.4)
	WM institution	3 (21.4)
	Pharmacy	3 (21.4)
	Public health centre	2 (14.3)
	Ministry of Health and Welfare	2 (14.3)
	Korea Consumer Agency	1 (7.1)
	KIDS	1 (7.1)
	MFDS	1 (7.1)

Why did you not report adverse events? (*n* = 32)	I did not know where to report	20 (62.5)
	I felt it was unnecessary	7 (21.9)
	I felt lazy	5 (15.6)

How did you deal with adverse events?^*∗*^ (*n* = 46)	Consulted with TKM doctors	20 (43.5)
	Nothing specific	13 (28.3)
	Requested refund	12 (26.1)
	Consulted with WM doctors	7 (15.2)
	Consulted with pharmacist	3 (6.5)

What did you think after the adverse events? (*n* = 46)	I need to see an expert	17 (37.0)
	Drugs can have adverse events and I am going to continue taking herbal medicines	14 (30.4)
	I cannot trust herbal medicines anymore and I am not going to take herbal medicines	13 (28.3)
	I do not know	2 (4.3)

^*∗*^Multiple responses possible. TKM: traditional Korean medicine; WM: western medicine; KIDS: Korea Institute of Drug Safety and Risk Management; MFDS: Ministry of Food and Drug Safety.

## Abbreviations

WHO: World Health Organization
T&CM: Traditional and Complementary Medicine
TKM: Traditional Korean medicine
GMP: Good Manufacturing Practice
MFDS: Ministry of Food and Drug Safety
KAERS: Korea Adverse Event Reporting System
KIDS: Korea Institute of Drug Safety and Risk Management.
