# Supplementary material for: Characteristics of Herbal Medicine Users and Adverse Events Experienced in South Korea: A Survey Study
Source: Evid Based Complement Alternat Med. 2017 Apr 10;2017:4089019. doi: 10.1155/2017/4089019 (PMC5402245; doi:10.1155/2017/4089019)
Supplement: Supplementary file 1 — The questionnaire aimed to investigate the usage experience of herbal medicines of general populations. It consists of two categories: (1) questions related to the herbal medicines usage over the past year and (2) questions related to adverse events experienced relating to the herbal medicines. [file 4089019.f1.docx]

Additional file 1: Questionnaire on usage experience of herbal medicines

Basic characteristics

·What is your sex and age?

1. Sex
2. men ② women
3. Age
4. 20-29 ②30-39 ③ 40-49 ④ 50-59 ⑤ 60-69

·What is your occupation?

1. Executive ② Professional ③ Office worker ④ Service worker

⑤ Sales worker ⑥ Agriculture, forestry and fishery worker ⑦ Craft worker

⑧ Mechanical technician ⑨ Simple labor worker ⑩ Soldier

⑪ Housewife ⑫ Student ⑬ Etc. ( ) ⑭ Unemployed

·What is the highest degree of education you have completed?

1. Middle school ② High school ③ College ④ Graduate school

1. Do you think that herbal medicine is safe?

① Yes, herbal medicine is safe. ② No, herbal medicine is not safe.

2. Have you taken herbal medicines during the last one year?

① Yes (→Go to No.3) ② No (→Go to No.11)

3.

1) During the last one year, where did you get your herbal medicines from? (Please select all that apply)

1. Korean Medicine hospital or clinic
2. Pharmacy
3. Oriental pharmacy
4. Traditional herb market
5. Health food store
6. Home-shopping
7. Hypermarket
8. Etc.

2) During the last one year, what type of herbal medicines have you taken? (Please select all that apply)

1. Herbal-drug prescribed by Korean medicine institutions
2. Insured powdery herbal medicines of KM institutions
3. Insured powdery herbal medicines of pharmacies
4. Decoction of Oriental pharmacy
5. Health functional food
6. Crude herb (used for cuisine or tea) health food store
7. Etc.

3) Why did you take your herbal medicine for the last one year? (Please select all that apply)

1. Treatment in traditional Korean medicine institutions
2. Health improvement
3. Recommendation from acquaintance
4. Recommendation from pharmacist
5. Etc.

4. Have you experienced any adverse event from your herbal medicines during the last one year?

① Yes (→Go to No.5) ② No (→End.)

5. What types of adverse events have you experienced? (Please select all that apply)

1. Digestive system
2. Nervous disorder
3. Skin
4. Musculoskeletal system
5. Respiratory system
6. Liver
7. Ear, nose, throat, and eye
8. Systemic disorder
9. Cardiovascular system
10. Blood system
11. Kidney
12. Urine system

6. How did you deal with the adverse events? (Please select all that apply)

1. Nothing specific
2. Consulted with traditional Korean medicine doctor
3. Consulted with western medicine doctor
4. Consulted with pharmacist
5. Requested for refund
6. Etc. ( )

7. What was your thought after the adverse events?

1. Drug can have adverse events and I am going to continue taking herbal medicines.
2. I cannot trust herbal medicines anymore and I am not going to take herbal medicines.
3. I need to see an expert.
4. I don’t know
5. Etc. ( )

8. Did you report the adverse events?

1. Yes (→Go to No.9) ② No (→Go to No.10)

9. To whom did you report the adverse events (Please select all that apply)

1. Traditional Korean Medicine institutions
2. Western medicine institutions
3. Pharmacy
4. Public health center
5. Ministry of Health and Welfare
6. Ministry of Food and Drug Safety
7. Korea Institute of Drug Safety and Risk Management
8. Korea Consumer Agency
9. Etc. ( ) → End.

10. Why did you not report the adverse events?

1. I felt it was unnecessary
2. I did not know where to report
3. I felt lazy
4. Ect. ( ) → End.

11. Why did you not take herbal medicines during the last one year? (Please select all that apply)

1. No necessity for medication
2. No effectiveness
3. Expensive price
4. Uncertainty of origins
5. Anxiety for harmful substances
6. Anxiety for possible adverse events
7. Disbelief in expiry date
8. Etc. → End.
